# Increased coagulation activity and genetic polymorphisms in the *F5, F10* and *EPCR* genes are associated with breast cancer: a case-control study

**DOI:** 10.1186/1471-2407-14-845

**Published:** 2014-11-19

**Authors:** Mari Tinholt, Marte Kathrine Viken, Anders Erik Dahm, Hans Kristian Moen Vollan, Kristine Kleivi Sahlberg, Øystein Garred, Anne-Lise Børresen-Dale, Anne Flem Jacobsen, Vessela Kristensen, Ida Bukholm, Rolf Kåresen, Ellen Schlichting, Grethe Skretting, Benedicte Alexandra Lie, Per Morten Sandset, Nina Iversen

**Affiliations:** Department of Medical Genetics, Oslo University Hospital and University of Oslo, Oslo, Norway; Department of Haematology, Oslo University Hospital, Oslo, Norway; Research Institute of Internal Medicine, Oslo University Hospital, Oslo, Norway; Institute of Clinical Medicine, University of Oslo, Oslo, Norway; Department of Immunology, Oslo University Hospital and University of Oslo, Oslo, Norway; Department of Genetics, Institute for Cancer Research, Oslo University Hospital, Oslo, Norway; The K.G. Jebsen Center for Breast Cancer Research, Faculty of Medicine, University of Oslo, Oslo, Norway; Department of Oncology, Oslo University Hospital Radiumhospitalet, Oslo, Norway; Department of Research, Vestre Viken, Drammen, Norway; Department of Pathology, Oslo University Hospital, Oslo, Norway; Department of Obstetrics and Gynecology, Oslo University Hospital, Oslo, Norway; Department of Clinical Molecular Biology (EpiGen), Akershus University Hospital, Lørenskog, Norway; Department of Breast and Endocrine Surgery, Oslo University Hospital, Oslo, Norway

**Keywords:** Tissue factor pathway, Single nucleotide polymorphisms, Breast cancer, Activated protein C resistance, D-dimer, Genotype-phenotype correlations, Factor V Leiden, Prothrombin G20210A, Hormone receptor status, Triple negative status

## Abstract

**Background:**

The procoagulant state in cancer increases the thrombotic risk, but also supports tumor progression. To investigate the molecular mechanisms controlling cancer and hemostasis, we conducted a case-control study of genotypic and phenotypic variables of the tissue factor (TF) pathway of coagulation in breast cancer.

**Methods:**

366 breast cancer patients and 307 controls were genotyped for SNPs (n = 41) in the *F2, F3* (TF)*, F5, F7, F10*, *TFPI* and *EPCR* genes, and assayed for plasma coagulation markers (thrombin generation, activated protein C (APC) resistance, D-dimer, antithrombin, protein C, protein S, and TF pathway inhibitor (TFPI)). Associations with breast cancer were evaluated using logistic regression to obtain odds ratios (ORs) and 95% confidence intervals (CIs), or the chi-square test.

**Results:**

Four SNPs in *F5* (rs12120605, rs6427202, rs9332542 and rs6427199), one in *F10* (rs3093261)*,* and one in *EPCR* (rs2069948) were associated with breast cancer. *EPCR* rs2069948 was associated with estrogen receptor (ER) and progesterone receptor (PR) positivity, while the SNPs in *F5* appeared to follow hormone receptor negative and triple negative patients. The prothrombotic polymorphisms factor V Leiden (rs6025) and prothrombin G20210A (rs1799963) were not associated with breast cancer. High APC resistance was associated with breast cancer in both factor V Leiden non-carriers (OR 6.5, 95% CI 4.1-10.4) and carriers (OR 38.3, 95% CI 6.2-236.6). The thrombin parameters short lag times (OR 5.8, 95% CI 3.7-9.2), short times to peak thrombin (OR 7.1, 95% CI 4.4-11.3), and high thrombin peak (OR 6.1, 95% CI 3.9-9.5) predicted presence of breast cancer, and high D-dimer also associated with breast cancer (OR 2.0, 95% CI 1.3-3.3). Among the coagulation inhibitors, low levels of antithrombin associated with breast cancer (OR 5.7, 95% CI 3.6-9.0). The increased coagulability was not explained by the breast cancer associated SNPs, and was unaffected by ER, PR and triple negative status.

**Conclusions:**

A procoagulant phenotype was found in the breast cancer patients. Novel associations with SNPs in *F5, F10* and *EPCR* to breast cancer susceptibility were demonstrated, and the SNPs in *F5* were confined to hormone receptor negative and triple negative patients. The study supports the importance of developing new therapeutic strategies targeting coagulation processes in cancer.

**Electronic supplementary material:**

The online version of this article (doi:10.1186/1471-2407-14-845) contains supplementary material, which is available to authorized users.

## Background

There is compelling evidence that blood coagulation and tumor biology are connected through multiple pathophysiological pathways. Disruption of the hemostatic balance is frequently observed in several cancer types [1–3]. The hypercoagulable state has been attributed to an adverse effect of malignant cells expressing procoagulants, but there is now evidence of a bidirectional interaction between cancer and coagulation [4]. Elevated plasma levels of activated coagulation markers, such as D-dimer, have been shown to be markers of cancer progression and poor outcome [5–7]. Plasma levels of the coagulation inhibitors antithrombin and protein C have been shown to decrease, while tissue factor (TF) pathway inhibitor (TFPI) was found to increase during cancer progression [8, 9], and several studies have shown that cancer patients acquire activated protein C (APC) resistance [2, 3, 10, 11].

During carcinogenesis a transcriptional program inducing expression of hemostatic genes is turned on [12]. Procoagulants produced by tumor- and stimulated host cells not only contribute to the increased risk of cancer-associated thrombosis, but can also trigger cancer- signaling pathways. Coagulation independent signaling increases the angiogenic and metastatic behavior of tumor cells and thereby accelerates the growth and spread of cancer. One of the most extensively studied procoagulants involved in cancer is TF. Induced by oncogenes, such as *K-ras* and the epidermal growth factor receptor (*EGFR*), TF is overexpressed in many cancers. TF expression has been shown to correlate with tumor progression and poor survival [13]. TF initiates the coagulation cascade by forming a complex with activated factor VII (FVIIa), which activates factor X (FX). The assembly of FXa with its activated cofactor, factor Va (FVa), leads to the generation of thrombin, fibrin formation and platelet activation. The activity of FXa and the TF/FVIIa catalytic complex is modulated by TFPI. In addition to coagulation activation, the TF complexes (TF/FVIIa and TF/FVIIa/FXa) may elicit G-protein coupled intracellular signaling mediated by protease- activated receptors (PARs). Activation of PAR-1 and PAR-2 results in expression of genes promoting angiogenesis, cell migration, proliferation, and metastasis [14]. Recently, it was demonstrated that the endothelial protein C receptor (EPCR) is able to bind the ternary TF/FVIIa/FXa complex and induce a more efficient PAR-1 and PAR-2 mediated signaling in endothelial cells [15]. EPCR positive breast cancer cells have an increased ability to form tumors *in vivo* [16].

The *F5* rs6025 and *F2* rs1799963 (commonly known as the factor V Leiden and the prothrombin G20210A polymorphisms, respectively) are well-established procoagulant polymorphisms that increase the risk of venous thrombosis, due to induction of APC resistance and increased levels of prothrombin, respectively. Mozsik *et al.* recently reported an association of factor V Leiden with gastrointestinal cancer [17], whereas Vossen *et al*. found a 6-fold increased risk of colorectal cancer for homozygous, but not for heterozygous factor V Leiden carriers [18]. Additional studies (across populations) on several cancer types have also failed to show an association with factor V Leiden heterozygotes [3, 19–22]. Except for Pihusch *et al.* [20], several studies have not been able to find an increased prevalence of the prothrombin G20210A polymorphism in cancer [3, 18, 19, 21, 22].

The *F7* gene polymorphism -402GA (rs510317) has been reported to be associated with breast cancer [23]. Still, limited information on the role of polymorphisms in hemostatic genes to cancer pathogenesis is available, in particular regarding the more common variants.

Breast cancer is a highly heterogenous disease with substantial variation at both the clinical and the molecular level. Immunohistochemical expression of the growth regulating hormone receptors; estrogen receptor (ER) and progesterone receptor (PR), in addition to overexpression and/or amplification of the oncogene human epidermal growth factor receptor 2 (HER2) are clinically relevant markers for prognostic and predictive purposes. The majority of breast tumors (~80%) show hormone receptor positivity and are likely to respond to endocrine (hormonal) therapy. 10-15% of breast cancers belong to a subgroup called triple negative breast cancers, defined by lack of ER, PR and HER2 overexpression. Triple negative breast cancers tend to have poor prognosis, and currently, no targeted therapy has been approved for this type of breast cancer [24].

In the present case-control study, we aimed to investigate the role of common single nucleotide polymorphisms (SNPs) in genes involved in the TF pathway of coagulation (i.e., the *F2, F3* (*TF*) *F5, F7, F10, EPCR,* and *TFPI* genes) on the susceptibility of breast cancer. In addition, markers of coagulation activity and plasma levels of coagulation inhibitors were measured, related to presence of breast cancer, and correlated to genotypes of breast cancer associated SNPs.

## Methods

### Patient material; cases and controls

The study comprised of 385 stage I or II female breast cancer patients (cases) enrolled between June 2008 and August 2010 at the Oslo University Hospital Ullevål, Oslo, and the Akershus University Hospital, Nordbyhagen, Norway. The cases were subjected to primary breast surgery (mastectomy or lumpectomy) without receiving any pre-operative treatment, and blood samples were drawn immediately before surgery. Cases that later were acknowledged to have metastatic disease were excluded. The controls comprised of 353 healthy women, who were originally recruited as controls in a study on the risk of venous thrombosis in pregnancy [25]. ER and PR status of the tumors were determined by immunohistochemistry and collected from pathology reviews, and tumor cell nuclei were scored according to pathology guidelines. HER2 status was determined by immunohistochemistry and/or by silver enhancement in situ hybridization (SISH) (Roche, Dual SISH HER-2) where a HER2 gene/centrosome 17 (CEP17) ratio of >2.2 defined HER2 positivity.

We excluded subjects who were not of Scandinavian descent (i.e., not from Norway, Sweden or Denmark) from genotypic and phenotypic analyzes, and subjects who were pregnant, or received anticoagulant- or hormone replacement therapy were excluded from the phenotypic analyzes. After excluding one case with metastases at the time of diagnosis, two cases that proved not to have breast cancer, and 16 non-Scandinavians, the final case group comprised of 366 breast cancer patients for both genotypic and phenotypic analyzes. Among the 353 control subjects, 46 were non-Scandinavian, thus leaving 307 controls for genotypic analyzes (i.e. SNPs). 34 controls were either pregnant or used oral contraceptives leaving 273 controls for phenotypic analyzes (i.e. hemostatic parameters). The average age at blood sampling was 57.7 (±11.2) (range 29-87) years for cases, and 40.0 (±5.6) (range 22-58) years for controls.

The Regional Committee for Medical and Health Research Ethics of South-East Norway approved the study (approval number 1.2006.1607, amendment 1.2007.1125 for Ullevål patients and 429-04148 for Akershus patients) and all included women gave their written informed consent to participate.

### Blood sampling

Venous blood samples were collected in Vacutainer vacuum tubes (Becton-Dickinson, Plymouth, UK) containing 0.5 mL buffered sodium citrate (0.129 mol/L). Whole blood was centrifuged for 15 min at 2000 g at room temperature within 1 hour to prepare platelet poor plasma, and aliquots were stored at -70°C until analyzed. Using the same blood collection procedure as described, plasma from 21 healthy subjects (9 men and 12 women, mean age 43 years) were collected to create a pooled normal plasma (PNP) reference. None of these 21 subjects had antithrombin-, protein C- or protein S deficiencies, were carriers for the factor V Leiden or the prothrombin G20210A polymorphisms, tested positive for antiphospholipid antibodies (lupus anticoagulant, or anticardiolipin- or anti-β2-glycoprotein 1 antibodies), and they did not use oral contraceptives or any other hormones.

### Phenotypic hemostatic parameters

The endogenous thrombin potential (ETP) was measured using the Calibrated Automated Thrombogram (CAT) assay [26], according to the manufacturer’s instructions (Thrombinoscope B.V, Maastricht, the Netherlands). Four thrombin generation parameters were recorded; ETP (time integral of the thrombin formation), lag time, peak thrombin (Peak), and time to peak thrombin (ttPeak). APC resistance was determined after the addition of APC (American Diagnostica Inc., Stamford, CT, USA), and the results were reported as APC-sensitivity ratio (APC-sr); which is the ratio of ETP in presence of APC divided by ETP in absence of APC normalized against the similar ratio obtained with PNP measured in the same run [25]. The coagulation inhibitors antithrombin and protein C activities, and free protein S antigen, were analyzed using the Chromogenix Coamatic® Antithrombin, the Chromogenix Coamatic® Protein C, and the Chromogenix Coamatic® Protein S-Free kits from Instrumentation Laboratory (Lexington, MA, USA). Free TFPI antigen and D-dimer were analyzed by the commercial enzyme-linked immunosorbent assay kits Asserachrom® Free TFPI and Asserachrom® D-DI from Diagnostica Stago, Asnières, France. The hemostatic parameters are shown in Additional file 1: Figure S1.

### DNA isolation and genotyping

DNA was either isolated on the BioRobot Universal with the QIAamp DNA Blood BioRobot MDx Kit (Qiagen, Hilden, Germany) and eluted in Qiagen buffer AE (10 mM Tris-Cl 0.5 mM EDTA; pH 9.0), or with the Gentra Autopure LS machine using the Puregene Genomic DNA purification Kit (Gentra Systems, Minneapolis, MN 55441 USA), or manually using the MasterPure TM DNA Purification Kit for Blood Version II (Epicentre® Biotechnologies, Madison, WI, USA). SNPs were genotyped with the iPLEX Gold massarray platform (Sequenom) at the Centre for Integrative Genetics, Norwegian University of Life Sciences, Ås, Norway.

### SNP selection and quality control

We used a SNP tagging approach to avoid genotyping redundant SNPs. By using a minor allele frequency (MAF) criterion of ≥10% and pairwise r^2^ ≥ 0.8 as a cut-off for proxies, 39 SNPs were selected in the following gene regions: *F2 (n = 3), F3* (*TF*) *(n = 4), F5 (n = 10), F7 (n = 2), F10 (n = 9), TFPI (n = 9)*, and *EPCR (n = 2)*. The tag-SNP selection was performed using the Tagger program ( http://www.broad.mit.edu/mpg/haploview/, [27]) implemented in Haploview v. 4.2 and genotype data from the Caucasian population (Utah residents with ancestry from northern and western Europe) from the HapMap project release 27, phase III on NCBI B36 assembly, dbSNPb126. Factor V Leiden (rs6025) and the prothrombin G20210A (rs1799963) polymorphisms were also included in the SNP selection. Hence, the final SNP selection consisted of 41 SNPs that were genotyped in both cases and controls.

Individuals with ≥50% missing genotypes and SNPs with <97% call rates were excluded for further analysis. SNPs that deviated from Hardy-Weinberg equilibrium in the exact test were also excluded (significance threshold *P* < 0.001).

One SNP failed genotyping in all individuals (*TFPI* rs3213739), two SNPs had genotyping call rates <97% (*F7* rs1475931 and *TFPI* rs2041778), and one SNP was out of Hardy Weinberg equilibrium in controls (*EPCR* rs867186). Hence, after filtering, 37 of the 41 genotyped SNPs remained for further analysis.

### Statistical methods

All statistical analyzes were performed using SPSS statistical software (version 21.0; SPSS Inc., Chicago, IL, USA) and PLINK v.1.07 (http://pngu.mgh.harvard.edu/~purcell/plink/).

Associations between each SNP and breast cancer were analyzed using an allelic chi-square (χ^2^) test with 1 degree of freedom. The false discovery rate (FDR) procedure described by Benjamini & Hochberg [28] was used to correct for multiple testing.

Odds ratios (ORs), 95% confidence intervals (CIs), and *P*-values were determined for the genotypes of the SNPs that were significant at the 5% level and had a FDR < 0.25 in the allelic test. Binary logistic regression under the additive risk model was applied with case/control status as the dependent variable, and genotypes (coded 0, 1, 2 for each extra risk allele) as the categorical independent variables. Risk alleles were defined as the alleles being more prevalent among cases, thus ORs >1 were obtained.

Independence between SNP associations was tested by conditional analysis in PLINK, where the allelic dosage for a given SNP was added as a covariate in a binary logistic regression model (additive model). The E-M algorithm was used to estimate haplotype frequencies, and haplotype-based association analysis was conducted using binary logistic regression (additive model). Haploview v. 4.2 was used for creating linkage disequilibrium (LD) plots, and the SNAP tool [29] was used to obtain pair-wise LD measurements. The Alamut software (v. 2.0) was used to predict if any of the associated SNPs, or their proxies, could affect splicing.

The plasma levels of hemostatic parameters were compared between cases and controls using t-test when normally distributed, or the non-parametric Mann-Whitney when the distribution was skewed. Tests with *P* < 0.05 were considered significantly different. Logistic regression was used to determine the associations with breast cancer status, or ER, PR, HR, and triple negative status, for either high or low levels of each of the hemostatic parameters. Case and control subjects were dichotomized according to either the 10^th^ or the 90^th^ percentiles of the hemostatic parameters’ plasma levels (defined in controls). The group with levels above the 10^th^ percentile or below the 90^th^ percentile served as the reference group.

Genotype-phenotype correlations were evaluated by the Kruskal-Wallis test. For tests with *P* < 0.05, follow-up pairwise comparisons were conducted using Mann Whitney U testing with Bonferroni correction, and genotype-phenotype pairs with at least one significant pairwise test were reported. For correlations with the APC resistant phenotype, factor V Leiden carriers were excluded due to the established role for the factor V Leiden variant in APC resistance.

## Results

### Associations between SNPs in TF pathway genes and risk of breast cancer

Associations between SNP alleles of the TF pathway genes and risk of breast cancer were assessed by comparing allele distributions between cases and controls (Table 1). A total of six SNPs in three distinct genes exhibited significantly different allele distributions with FDR < 0.25 (four in *F5*: rs12120605 (*P =* 0.026), rs6427202 (*P* = 0.028), rs9332542 (*P* = 0.023), rs6427199 (*P* = 0.037), one in *F10*: rs3093261 (*P* = 0.011), and the one in *EPCR*: rs2069948 (*P* = 0.030)).

**Table 1:** **Allele distributions of SNPs in TF pathway genes in cases and controls**

Gene	Chr	SNP	Bp (hg18)	Region	Minor allele	Major allele	Freqency cases	Frequency controls	χ ^2^	OR	*P*-value (unadj.)	FDR
*F2*	11	rs2070852	46701501	Intronic	C	G	0.290	0.316	0.99	0.89	0.319	0.83
*F2*	11	rs3136516	46717332	Intronic	G	A	0.448	0.448	0.00	1.00	0.996	1.00
*F2*	11	rs5896	46701579	Coding (T > M)	T	C	0.133	0.127	0.09	1.05	0.760	0.93
*F2*	11	*rs1799963*	*46717631*	*3UTR*	*A*	*G*	*0.013*	*0.005*	*2.20*	*2.60*	*0.138*	*0.61*
*F3*	1	rs3917643	94774455	Intronic	C	T	0.058	0.080	2.58	0.70	0.109	0.57
*F3*	1	rs696619	94777808	Intronic	A	G	0.414	0.440	0.87	0.90	0.351	0.83
*F3*	1	rs1324214	94769876	Intronic	A	G	0.240	0.271	1.59	0.85	0.207	0.70
*F3*	1	rs3917615	94774578	Intronic	T	C	0.439	0.433	0.04	1.02	0.839	0.93
***F5***	**1**	**rs12120605**	**167789178**	**Intronic**	T*	**G**	**0.140**	**0.100**	**4.93**	**1.47**	**0.026**	**0.22**
***F5***	**1**	**rs6427202**	**167795454**	**Intronic**	C*	**T**	**0.454**	**0.394**	**4.82**	**1.28**	**0.028**	**0.22**
***F5***	**1**	**rs9332542**	**167805907**	**Intronic**	**A**	G*	**0.286**	**0.344**	**5.16**	**0.76**	**0.023**	**0.22**
***F5***	**1**	**rs6427199**	**167790161**	**Intronic**	**A**	G*	**0.364**	**0.420**	**4.36**	**0.79**	**0.037**	**0.23**
*F5*	1	rs6012	167795204	Intronic	T	C	0.162	0.164	0.01	0.98	0.906	0.93
*F5*	1	rs4524	167778379	Coding (K > R)	C	T	0.261	0.257	0.03	1.02	0.871	0.93
*F5*	1	rs4656687	167771782	Intronic	C	T	0.323	0.312	0.17	1.05	0.676	0.93
*F5*	1	*rs6025*	*167785673*	*Coding (Q > R)*	*T*	*C*	*0.033*	*0.034*	*0.02*	*0.96*	*0.900*	*0.93*
*F5*	1	rs9287095	167805090	Intronic	A	G	0.094	0.104	0.34	0.90	0.561	0.93
*F5*	1	rs10158595	167786988	Intronic	T	C	0.223	0.248	1.14	0.87	0.286	0.83
*F5*	1	rs9332618	167767105	Intronic	A	G	0.133	0.139	0.08	0.95	0.773	0.93
*F7*	13	rs491098	112817347	Intronic	C	G	0.106	0.117	0.39	0.90	0.530	0.93
***F10***	**13**	**rs3093261**	**112824083**	**Near 5UTR**	T*	**C**	**0.460**	**0.391**	**6.41**	**1.33**	**0.011**	**0.22**
*F10*	13	rs3211744	112832999	Intronic	T	G	0.136	0.154	0.85	0.87	0.358	0.83
*F10*	13	rs2026160	112840894	Intronic	C	A	0.284	0.258	1.08	1.14	0.298	0.83
*F10*	13	rs9549675	112846885	Intronic	T	C	0.205	0.239	2.10	0.83	0.148	0.61
*F10*	13	rs3211719	112825510	Intronic	G	A	0.252	0.245	0.09	1.04	0.769	0.93
*F10*	13	rs3211752	112835460	Intronic	G	A	0.499	0.489	0.13	1.04	0.716	0.93
*F10*	13	rs556694	112828042	Intronic	C	T	0.093	0.089	0.04	1.04	0.836	0.93
*F10*	13	rs3211770	112841850	Intronic	A	G	0.109	0.113	0.05	0.96	0.818	0.93
*F10*	13	rs473598	112849190	Intronic	A	G	0.128	0.137	0.23	0.92	0.629	0.93
***EPCR***	**20**	**rs2069948**	**33226150**	**Intronic**	C*	**T**	**0.468**	**0.408**	**4.72**	**1.27**	**0.030**	**0.22**
*TFPI*	2	rs2192825	188099064	Intronic	C	T	0.439	0.402	1.81	1.16	0.178	0.66
*TFPI*	2	rs7594359	188117093	Intronic	T	C	0.461	0.441	0.52	1.08	0.472	0.93
*TFPI*	2	rs2192824	188077036	Intronic	T	C	0.449	0.428	0.54	1.09	0.461	0.93
*TFPI*	2	rs13424790	188032097	Downstream	G	T	0.317	0.310	0.08	1.03	0.778	0.93
*TFPI*	2	rs8176548	188048580	Intronic	T	C	0.351	0.347	0.02	1.02	0.878	0.93
*TFPI*	2	rs10187622	188122406	Intronic	T	C	0.161	0.165	0.03	0.97	0.864	0.93
*TFPI*	2	rs12613071	188096556	Intronic	C	T	0.202	0.195	0.09	1.04	0.762	0.93

*P*-values were determined by the χ^2^ -test. Alleles for the positive DNA strand (UCSC annotated) are shown. Significantly associated SNPs; **bold**, and factor V Leiden (*F5* rs6025) and prothrombin G20210A (*F2* rs1799963); *italic*. *Risk alleles for significant SNPs.

Chr: chromosome. OR: Odds ratio as determined for the minor allele with the major allele as reference. FDR: False discovery rate as described by Benjamini and Hochberg [28].

The genotype distributions for these six SNPs were compared using an additive model, since the underlying genetic model was unknown. All loci showed significant associations also at the genotypic level, with ORs ranging from 1.27 to 1.49 (Additional file 2: Table S1).

Furthermore, whereas the association with *EPCR* rs2069948 was restricted to patients with ER and PR positive tumors (OR 1.27, 95% CI 1.01-1.58; and OR 1.30, 95% CI 1.03-1.65, respectively), there was an overall trend that the four SNPs in *F5* were confined to hormone receptor negative patients (ER/PR negative) (ORs from 1.54 to 1.99) and triple negative patients (ER/PR/HER2 negative) (ORs from 1.68 to 2.11) when compared with healthy controls (Additional file 3: Table S2). However, the analyzes lacked power to show significant differences in genotype distributions between patient subgroups.

The allele distribution for factor V Leiden was equal among cases (3.3%) and controls (3.4%), while the prothrombin G20210A polymorphism appeared more frequently (non-significant) in patients (1.3%) compared to controls (0.5%) (Table 1). Only heterozygous carriers of either polymorphism were detected.

According to the SNAP tool, each of the *F5* rs6427202, *F5* rs9332542, and *EPCR* rs2069948 SNPs were all in strong or perfect LD (r^2^ = 0.93-1.00) with several SNPs in the European (CEU) population. The *F5* rs6427202 SNP was in strong LD (r^2^ ≥ 0.96) with 20 intronic *F5* SNPs, but also with five SNPs in the P-selectin coding gene; *SELP* (r^2^ ≥ 0.93)*.* Moreover, in the Regulome database (RegulomeDB) [30], the *F5* rs9332542 and four SNPs in perfect LD (rs2227245, rs2213872, rs2213873, rs6662176) were annotated as cis-acting expression quantitative trait loci (eQTL) for *F5*, and the *F10* rs3093261 was predicted to be an eQTL for the *LAMP1* gene encoding lysosome-associated membrane protein 1 (LAMP-1), located ~175 kb downstream of *F10* rs3093261. Using the Alamut software, none of the SNPs within a 300 bp distance from the nearest splice site, appeared to affect splicing.

### Conditional- and haplotype analysis of the *F5*SNPs associated with breast cancer

**Figure 1:**
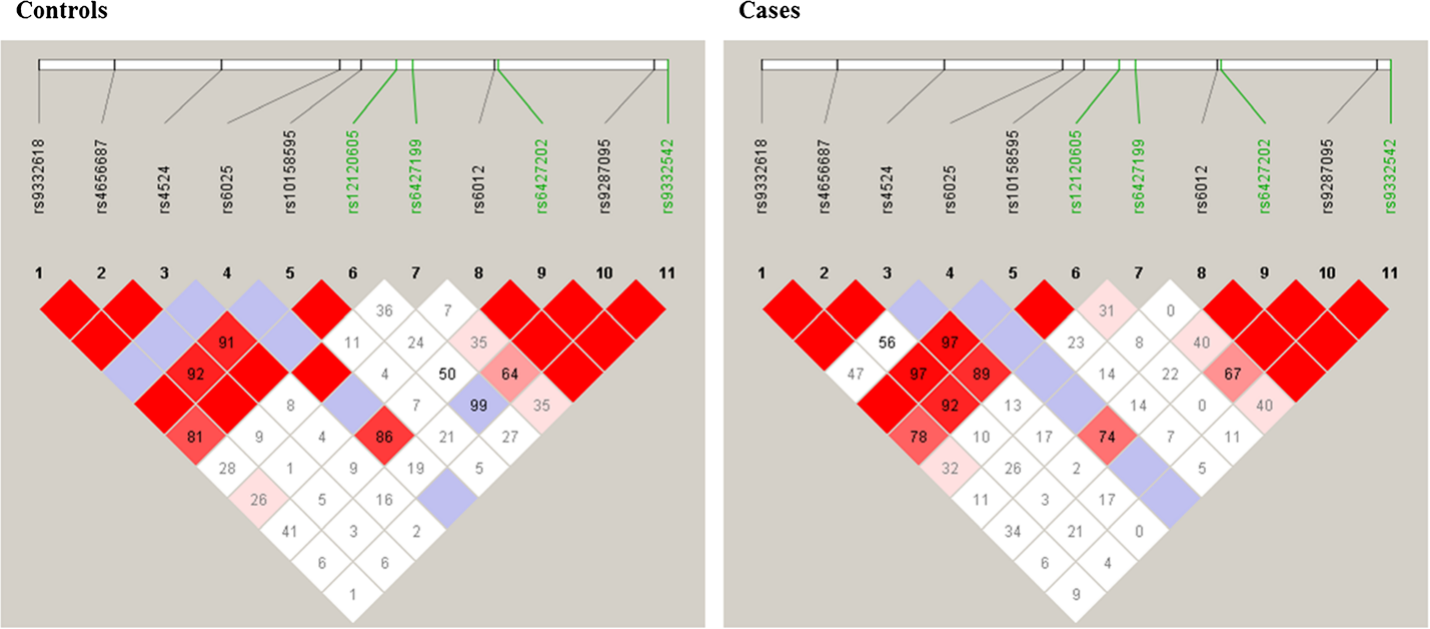
**Linkage disequilibrium (LD) plots of the** ***F5*** **SNPs.** Linkage disequilibrium (LD) plots of the 11 analyzed SNPs including factor V Leiden (rs6025), within the *F5* gene for controls (left plot) and cases (right plot). The LD measure D’ is shown. The disease associated SNPs are depicted in green.

In total, four breast cancer associated SNPs were found in the *F5* gene region (rs12120605, rs6427202, rs9332542, and rs6427199), and the interdependence of these SNPs on breast cancer risk was investigated (Table 2). The rs12120605 appeared to represent an independent signal, as this SNP remained significantly associated after conditioning on the other three *F5* SNPs as separate covariates. Moreover, when set as the conditional SNP, the rs12120605 did not diminish the significance of the other three SNPs, reflecting the modest pairwise LD with these SNPs (D’ ≤ 0.50) (Figure 1). In contrast, a dependency appeared to exist between the rs6427202, rs9332542, and rs6427199 as their effects were neutralized when conditioned on each other. This result, combined with the LD structure between the three SNPs (D’ between 0.35-1.00) (Figure 1), pointed towards a potential haplotype effect. Indeed, the haplotype consisting of all three individual risk alleles (C-G-G) was common in the population (frequency of 0.32), and was found to be significantly associated with breast cancer (OR 1.39, *P* = 0.011). Conditioning on factor V Leiden did not alter the association of any of the other *F5* SNPs (data not shown).

**Table 2:** **Results of the conditional association analysis for the four significant** ***F5*** **gene SNPs**

SNP	Original ORs (*P*-values)	ORs (*P*-values) conditioned on rs12120605	ORs (*P*-values) conditioned on rs6427202	ORs (*P*-values) conditioned on rs9332542	ORs (*P*-values) conditioned on rs6427199
**rs12120605**	1.49 (0.024)	-	*1.55 (0.015)*	*1.54 (0.016)*	*1.62 (0.009)*
**rs6427202**	1.32 (0.021)	*1.36 (0.010)*	-	1.19 (0.218)	1.24 (0.085)
**rs9332542**	1.32 (0.022)	*1.35 (0.014)*	1.20 (0.244)	-	1.23 (0.095)
**rs6427199**	1.27 (0.036)	*1.33 (0.014)*	1.22 (0.111)	1.20 (0.141)	-

ORs and *P*-values are shown before and after conditioning on each of the SNPs. Significant conditional associations are shown in *italic.*

### Relation between hemostatic parameters and breast cancer

Coagulation activity and levels of coagulation inhibitors were compared between cases and controls to explore if any hemostatic abnormalities existed. Median levels and *P*-values are provided in Additional file 4: Table S3.

The estimated associations between breast cancer status and either high or low levels of the hemostatic parameters are shown in Table 3. From the CAT assay, an association with breast cancer was predicted for lag times and times to peak thrombin below the 10^th^ percentile, and for peak thrombin above the 90^th^ percentile, with ORs ranging from 5.8 to 7.1. ETP above the 90^th^ percentile did not associate with breast cancer. APC resistance levels above the 90^th^ percentile associated with breast cancer in factor V Leiden non-carriers (OR 6.5, 95% CI 4.06-10.35), but also in factor V Leiden carriers (OR 38.3, 95% CI 6.2-236.6). Moreover, subjects with high D-dimer levels (>90^th^ percentile) were also associated with breast cancer disease (OR 2.0, 95% CI 1.26-3.28). No association with breast cancer was found for low levels of the coagulation inhibitors protein C, protein S or free TFPI, but for antithrombin activity below the 10^th^ percentile, the association with breast cancer was ~6-fold higher compared to activity above the 10^th^ percentile. None of the associated hemostatic parameters were specific to the different subsets of patients, as defined by the ER and PR hormone receptor status or triple negative status (Additional file 5: Table S4).

**Table 3:** **Distribution of cases and controls among the high or low level categories of the hemostatic parameters**

Hemostatic parameter	Cases (n)	Controls (n)	OR	95% CI	*P*-value
**Coagulation activity:**					
ETP (%)*					
<90th percentile	309	246	Ref.	Ref.	Ref.
>90th percentile	37	27	1.09	0.65-1.84	0.744
Lag time (%)*					
>10th percentile	211	246	Ref.	Ref.	Ref.
<10th percentile	135	27	5.83	3.71-9.16	<0.001
ttPeak (%)*					
>10th percentile	206	249	Ref.	Ref.	Ref.
<10th percentile	140	24	7.05	4.40-11.29	<0.001
Peak (%)*					
<90th percentile	208	246	Ref.	Ref.	Ref.
>90thpercentile	138	27	6.05	3.85-9.50	<0.001
**APC resistance (nAPC-sr)**					
*FV Leiden non-carriers*					
<90th percentile	190	228	Ref.	Ref.	Ref.
>90th percentile	135	25	6.48	4.06-10.35	<0.001
*FV Leiden carriers*					
<90th percentile	4	18	Ref.	Ref.	Ref.
>90th percentile	17	2	38.3	6.2-236.6	<0.001
**D-dimer (ng/mL)**					
<90th percentile	292	246	Ref.	Ref.	Ref.
>90th percentile	65	27	2.03	1.26-3.28	0.004
**Coagulation inhibitors:**					
**AT (%)**					
>10th percentile	215	246	Ref.	Ref.	Ref.
<10th percentile	135	27	5.72	3.64-8.99	<0.001
**Protein C (%)**					
>10th percentile	310	246	Ref.	Ref.	Ref.
<10th percentile	40	27	1.18	0.702-1.97	0.539
**Protein S (%)**					
>10th percentile	325	246	Ref.	Ref.	Ref.
<10th percentile	22	27	0.62	0.34-1.11	0.106
**Free TFPI (ng/mL)**					
>10th percentile	319	246	Ref.	Ref.	Ref.
<10th percentile	39	27	1.11	0.66-1.87	0.683

ORs, 95% CI and *P*-values were obtained by logistic regression with respect to cases.

AT = antithrombin. ttpETP = time to thrombin peak. nAPC-sr = normalised APC sensitivity ratio. (%) describes activity as compared to pooled normal plasma (PNP). *CAT-assay variables.

Since increased APC resistance in cancer has been detected in several previous studies, adjustments were made for the hemostatic parameters that correlated to APC resistance; protein C (ρ = -0.18, *P* = 0.003), protein S (ρ = -0.33, *P* < 0.001), and free TFPI (ρ = -0.42, *P* < 0.001). Adjusting for each of these parameters as separate covariates had only a modest impact on the association between high APC resistance and breast cancer in factor V Leiden non-carriers (data not shown), and the OR obtained in the full model with all covariates included (OR 8.6, 95% CI 5.1-14.3), was similar to that of the unadjusted model in Table 3 (OR 6.5, 95% CI 4.06-10.35). Equivalent results were obtained for factor V Leiden carriers (adjusted OR 50.7, 95% CI 6.6-390.9 vs. unadjusted OR 38.3, 95% CI 6.2-236.6 (Table 3)). Age did not correlate to any of the associated coagulation parameters (assessed in controls), except for an inverse correlation to APC resistance (ρ = -0.13, *P* = 0.032).

### Genotype-phenotype associations

**Figure 2:**
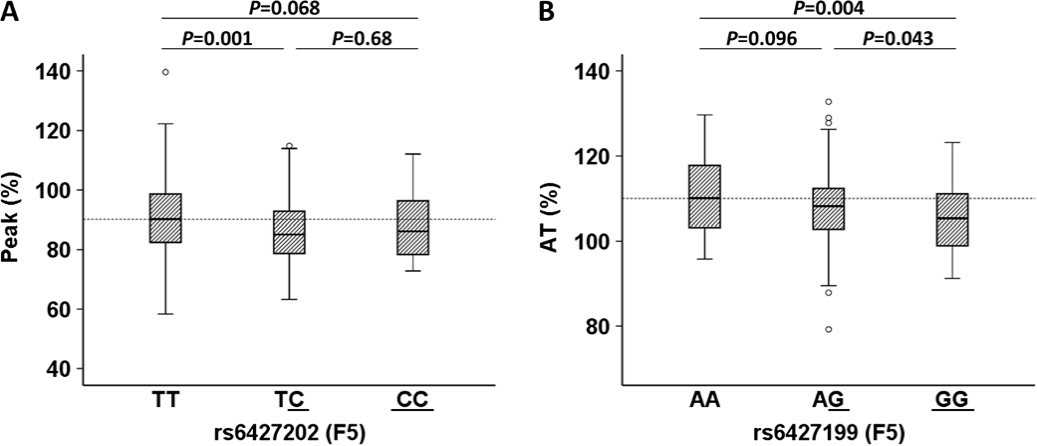
**Significant genotype-phenotype correlations.** Significant genotype-phenotype correlations in the control group after pairwise comparison analyzed by the Mann Whitney U test. Tests with ≥1 significant Bonferroni corrected *P*-value(s) are shown. (Bonferroni corrected *P*; 0.05/9 = 0.006). Distribution of **(A)** Peak (%) across the genotypes of *F5* rs6427202, and **(B)** antithrombin (%) across the genotypes of *F5* rs6427199. The box and whiskers plots show the minimum, 25th percentile, median, 75th percentile and maximum levels of the hemostatic parameters plotted against the genotype of the SNPs. The risk alleles of the SNPs are underlined.

In an effort to explain some of the hemostatic alterations observed in the patients, we searched for possible regulatory relationships between the genetic variations and the hemostatic parameters. Only the hemostatic parameters being significantly altered were considered, and we explored if any of these parameters were unevenly distributed across the genotypes of the six breast cancer associated SNPs in Table 1 (and Additional file 2: Table S1). Genotype-phenotype associations were made separately for controls and cases since divergent regulatory mechanisms could exist in the two groups. Two significant correlations were found in the control group; *F5* rs6427202 correlated with thrombin peak (Figure 2A), whereas *F5* rs6427199 correlated with antithrombin (Figure 2B). Because high thrombin peak and low antithrombin activity were found associated with breast cancer (Table 3), we adjusted for *F5* rs6427202 and *F5* rs6427199, respectively. However, these adjustments did not change the OR estimates obtained in the unadjusted model (data not shown). Interestingly, a trend for a correlation between the number of *F5* rs6427199 risk alleles and high APC resistance was found in controls (*P* = 0.15). *F5* rs6427199 may thus be an interesting candidate for general investigations of novel APC resistance inducing factors. No genotype-phenotype correlations were found in the case group.

## Discussion

Hypercoagulability is a common, but complex and multifactorial phenomenon in cancer. Although involvement of both clinical and biological aspects is recognized, the precise mechanism(s) underlying how the hemostatic system relates to cancer is not clear.

Among the 37 common SNPs in seven TF pathway genes (*TF, F2, F5, F7, F10, TFPI* and *EPCR*), six SNPs in three separate genes were found to be associated with breast cancer: four intronic SNPs in the *F5* gene (rs12120605, rs6427202, rs9332542 and rs6427199), one in the upstream region of the *F10* gene (rs3093261), and one intronic SNP in the *EPCR* gene (rs2069948). This is first-time evidence for an association between these SNPs and cancer.

Since breast cancer is a heterogeneous disease, certain risk factors may be specific for subsets of patients. In this study, the SNP in *EPCR* was associated with patients positive for ER and PR, while the SNPs in *F5* showed a tendency towards a preferential association with hormone receptor negative patients and triple negative patients. This might indicate that the *F5* SNPs may influence breast cancer etiology in hormone receptor negative/triple negative patients.

The association with *F5* expression [31] represents a possible link to the increased coagulation activity in breast cancer, and *F10* rs3093261, located between the *F10* and *F7* gene, has been associated with increased FVII levels in stroke patients [32], which may link *F10* rs3093261 to the increased coagulation activation observed in our study.

Supporting a role in cancer, EPCR has shown tumor growth promoting effects in mice [16].

Notably, the associated *F5* SNPs were common variants, and were independent of factor V Leiden carrier status. We found no association with factor V Leiden heterozygosity. Although not previously investigated in untreated breast cancer, studies in gastric- [19], gynaecological- [21], colorectal- [3], and oral cancer [22, 33] support this lack of association. So far, the study by Vossen *et al.* is the only study large enough to investigate the significance of homozygous factor V Leiden, reporting a 6-fold increased risk of colorectal cancer in homozygous carriers [18]. Interestingly, a reduced cancer risk (~30%) for heterozygous factor V Leiden carriers was reported in the same study [18]. Neither did we find a significant association with the prothrombin G20210A variant. However, given the low allele frequency (0.5-1.3%), a larger sample size would be needed to establish the role of this variant in breast cancer risk. Correspondingly, other studies across cancer types did not succeed in finding an association with prothrombin G20210A [3, 19, 21, 22, 33]. Pihusch *et al.* found an increased risk of gastrointestinal cancer for this variant [20], while Vossen *et al.* found a decreased risk of colorectal cancer for prothrombin G20210A heterozygotes [18].

In addition to the SNP discoveries, our study also provides evidence of a hypercoagulable state in the breast cancer patients, as detected by the CAT assay and increased APC resistance and D-dimer. The CAT assay parameters lag time, time to peak thrombin and peak thrombin were all associated with breast cancer, but not total ETP. Hence, the combination of the kinetic CAT parameters is likely to provide a better understanding of hypercoagulable states than do individual parameters [34]. Although care should be taken in comparing different thrombin assays [35], our CAT assay results are in line with previous studies in breast cancer reporting increased levels of thrombin formation [2], and shortened activated partial thromboplastin times (aPTT) [36].

We also found that increased APC resistance was associated with breast cancer. This finding is supported by a former breast cancer study [2], and studies in colorectal- [3] and gastrointestinal cancer [10]. In line with the existing literature, levels of protein S, TFPI, and also protein C correlated inversely to APC resistance [2, 37]. However, these potential APC resistance determinants did not affect the association between APC resistance and breast cancer. Interestingly, we found that increased APC resistance was associated with breast cancer in both carriers and non-carriers of factor V Leiden. Supported by previous studies [1, 38], this suggests that the acquired APC resistant phenotype appears more crucial in cancer than APC resistance caused by factor V Leiden. Acquired APC resistance could be due to increased levels of coagulation factors like factor V and factor VIII [11], yet unidentified factors produced by tumor- or stimulated host cells, or novel hereditary causes. Of note, the factor V Cambridge (rs118203906) variant, previously associated with increased APC resistance [39], turned out to be monomorphic in our case subjects (data not shown).

Further demonstrating the procoagulant state, high levels of the fibrin degradation product D-dimer were also associated with breast cancer. This confirms previous studies in breast cancer [6, 36, 40], and also other cancers like colorectal- [41], lung- [42] and gastric cancer [43]. These studies also demonstrated D-dimer as a potential important marker in disease stage prediction and for prognostic purposes. Furthermore, increased levels of D-dimer in cancer have been detected even in the absence of thrombosis [44].

Among the coagulation inhibitors, only low levels of antithrombin were associated with breast cancer. Decreased antithrombin levels have previously been found in breast cancer by Nijziel *et al.* [2], while no difference was observed either in another breast cancer study [45], a study on colorectal- [3] or a study on advanced cancers [1]. Supporting our observations, the two latter studies also found that neither protein S nor protein C levels in patients deviated from healthy controls. In a study of gastrointestinal cancer, Lindahl *et al.* found that the activity of antithrombin and protein C decreased [8], while the TFPI activity increased as the cancer progressed. Later, Iversen *et al.* confirmed that the median levels of TFPI activity were above the upper normal limit in gastrointestinal- and lung cancer, and in metastatic patients. In contrast, median TFPI activity in breast cancer was within the normal range [9]. Comparable to the latter, we found no difference in levels of TFPI in the cases compared to controls. Thus, the TFPI levels may vary according to cancer type and disease stage.

Noteworthy, the hypercoagulability did not seem to depend on hormone receptor status, as the estimated associations between breast cancer and the hemostatic plasma markers were not significantly different between hormone receptor negative or triple negative patients and patients with other subtypes. This observation is in agreement with a recent breast cancer study that did not observe any variation in D-dimer, prothrombin times (PT), and aPTT according to ER or PR status [36].

Since breast cancer is among the cancers with the lowest risk of thrombosis [46], our study indicates that activated coagulation may reflect the biology of the underlying tumor and could be an important indicator of cancer progression. In this context, it should be emphasized that the herein retrospective data is only suited to assess the presence of a hypercoagulable state in patients already diagnosed with breast cancer. Prospective studies are needed to establish whether coagulation activation precedes breast tumor development, or if it mirrors the course of the established disease. Interestingly, a prospective study reported a ~3-fold increased rate of digestive tract cancers in men with persistent coagulation activation [47].

The present work represents the most comprehensive study to investigate hemostasis in a homogenous (Scandinavian) breast cancer material. The tag SNP selection ensures good coverage of the normal genetic diversity within each selected gene. In this respect, it should be noted that our SNP associations may be a reflection of LD with other known or yet unknown true causal variants. In order to verify the significance of the associated SNPs, our findings should be validated in a different study population and other cancer types.

One limitation of the study is that the breast cancer patients were older than the control women, thus, an age-related bias could exist for the comparison of hemostatic plasma markers between patients and controls. Although supported by the existing literature, these results should therefore be interpreted with some caution. On the other hand, the inclusion criteria eliminate a possible effect of anticoagulant- or hormone therapy, and pregnancy, as well as chemotherapy, on the hemostatic parameters.

## Conclusions

This study has established the existence of a global procoagulant profile in breast cancer patients. The coagulation activity seemed to be independent of factor V Leiden and prothrombin G20210A. Instead, novel associations between common SNPs in genes of the TF pathway (*F5, F10,* and *EPCR*) and breast cancer susceptibility were demonstrated. Based on both phenotypic and genotypic evidence, this study supports the importance of developing new therapeutic strategies targeting coagulation processes in cancer.

## Electronic supplementary material

Additional file 1: Figure S1: TF-pathway of coagulation. The TF-FVIIa complex initiates the coagulation cascade by activating FX to FXa, which aided by its cofactor FVa cleaves prothrombin to generate thrombin. Thrombin cleaves fibrinogen to form fibrin monomers that together with activated platelets form a blood clot. D-dimer is a fibrin degradation product. Coagulation inhibitors and their targets are designated. EPCR has been indicated to associate with the TF-FVIIa complex. The FVL (rs6025) polymorphism prevents the ability of aPC to cleave and inhibit FVa, causing (inherited) APC resistance. The PT G20210A (rs1799963) polymorphism increases the rate of prothrombin protein production. Regular arrows and blunt-end arrows illustrate activation and inhibition, respectively. The hemostatic markers measured in plasma in this study are shaded in grey. EPCR = endothelial protein C receptor, TF = tissue factor, FVIIa = activated factor VII, FX = factor X, FXa = activated factor X, FVa = activated factor Va, TFPI = tissue factor pathway inhibitor, PS = protein S, aPC = activated protein C, AT = antithrombin, PT = prothrombin, FVL = factor V Leiden, APCR = activated protein C resistance. (PDF 160 KB)

Additional file 2: Table S1: Genotype distributions of the significant SNPs in TF pathway genes in cases and controls. ORs, 95% CI and *P*-values determined with respect to the risk allele (bold) using logistic regression (additive model). Alleles for the positive DNA strand (UCSC annotated) are shown. (PDF 100 KB)

Additional file 3: Table S2: The breast cancer associated SNPs stratified by hormone receptor status (ER and PR) and triple negative status (ER negative/PR negative/HER2 negative) (additive model in binary logistic regression). ORs determined with respect to the risk allele. Significant associations are shown in bold. (PDF 188 KB)

Additional file 4: Table S3: Plasma levels of hemostatic parameters in cases and controls. Median values with IQR shown in brackets. (PDF 90 KB)

Additional file 5: Table S4: ORs and *P*-values for hemostatic parameters stratified by hormone receptor status (ER and PR) and triple negative status (ER negative/PR negative/HER2 negative). (PDF 99 KB)

## Abbreviations

TF: Tissue factor
APC: Activated protein C
TFPI: Tissue factor pathway inhibitor
ER: Estrogen receptor
PR: Progesterone receptor
HR: Hormone receptor
OR: Odds ratio
EPCR: Endothelial protein C receptor
HER2: Human epidermal growth factor receptor 2
SNP: Single nucleotide polymorphism
ETP: Endogenous thrombin potential
CAT: Calibrated automated thrombogram
ttPeak: Time to Peak
MAF: Minor allele frequency
FDR: False discovery rate
CI: Confidence interval
LD: Linkage disequilibrium.
