# Supplementary figures and images for: Increased coagulation activity and genetic polymorphisms in the F5, F10 and EPCR genes are associated with breast cancer: a case-control study
Source: BMC Cancer. 2014 Nov 19;14:845. doi: 10.1186/1471-2407-14-845 (PMC4251949; doi:10.1186/1471-2407-14-845)

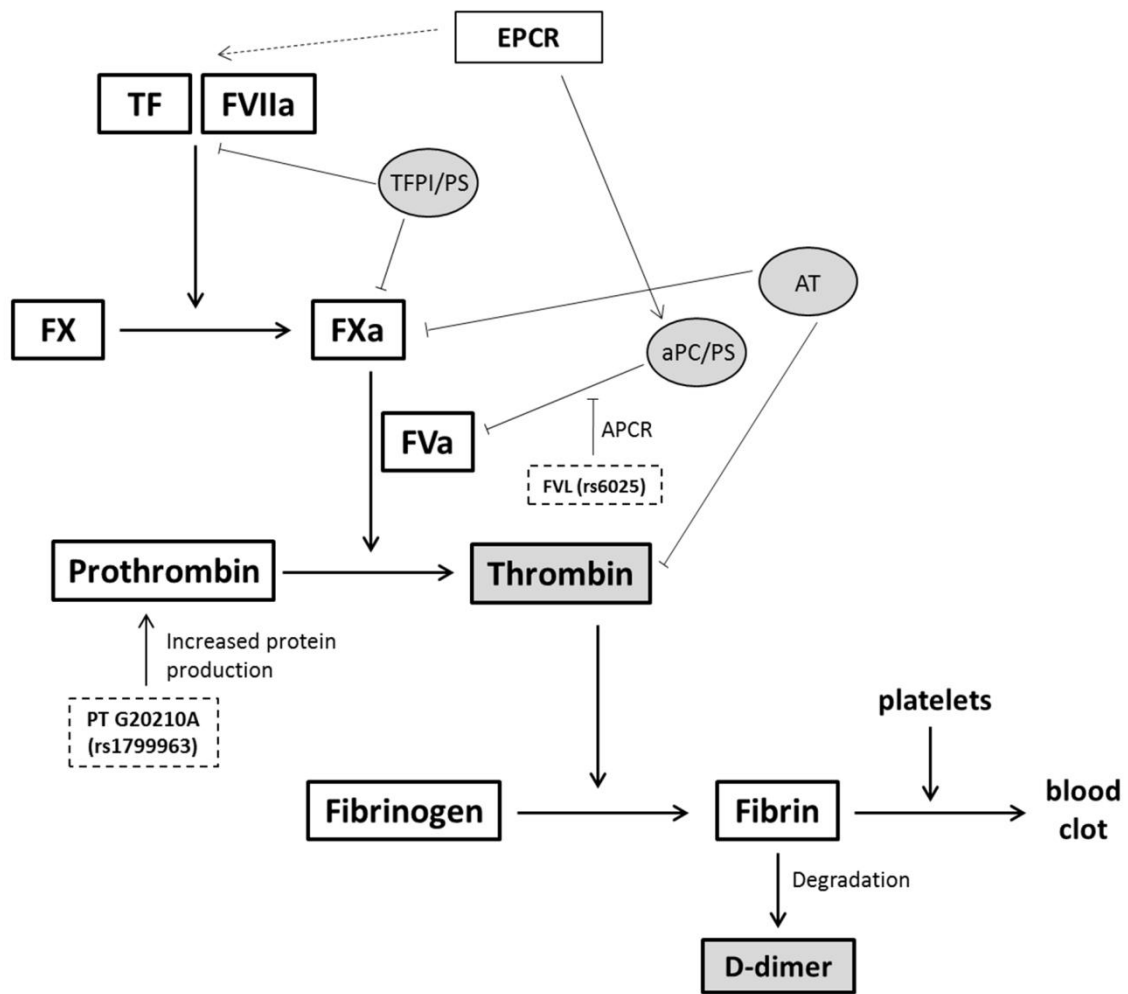

Supplement: Supplementary file 1 — Additional file 1: Figure S1: TF-pathway of coagulation. The TF-FVIIa complex initiates the coagulation cascade by activating FX to FXa, which aided by its cofactor FVa cleaves prothrombin to generate thrombin. Thrombin cleaves fibrinogen to form fibrin monomers that together with activated platelets form a blood clot. D-dimer is a fibrin degradation product. Coagulation inhibitors and their targets are designated. EPCR has been indicated to associate with the TF-FVIIa complex. The FVL (rs6025) polymorphism prevents the ability of aPC to cleave and inhibit FVa, causing (inherited) APC resistance. The PT G20210A (rs1799963) polymorphism increases the rate of prothrombin protein production. Regular arrows and blunt-end arrows illustrate activation and inhibition, respectively. The hemostatic markers measured in plasma in this study are shaded in grey. EPCR = endothelial protein C receptor, TF = tissue factor, FVIIa = activated factor VII, FX = factor X, FXa = activated factor X, FVa = activated factor Va, TFPI = tissue factor pathway inhibitor, PS = protein S, aPC = activated protein C, AT = antithrombin, PT = prothrombin, FVL = factor V Leiden, APCR = activated protein C resistance. (PDF 160 KB) [file 12885_2014_5043_MOESM1_ESM.pdf]
