# Supplementary material for: Increased coagulation activity and genetic polymorphisms in the F5, F10 and EPCR genes are associated with breast cancer: a case-control study
Source: BMC Cancer. 2014 Nov 19;14:845. doi: 10.1186/1471-2407-14-845 (PMC4251949; doi:10.1186/1471-2407-14-845)
Supplement: Supplementary file 2 — Additional file 2: Table S1: Genotype distributions of the significant SNPs in TF pathway genes in cases and controls. ORs, 95% CI and P-values determined with respect to the risk allele (bold) using logistic regression (additive model). Alleles for the positive DNA strand (UCSC annotated) are shown. (PDF 100 KB) [file 12885_2014_5043_MOESM2_ESM.pdf]

Genotype distributions of the significant SNPs in TF pathway genes in cases and controls. ORs, 95% CI and *P*-values determined with respect to the risk allele (**bold**) using logistic regression (additive model). Alleles for the positive DNA strand (UCSC annotated) are shown.

SNP	Cases (n)	Controls (n)	OR	95% CI	*P*-value (unadj)
**rs12120605 (*F5*)**					
GG	262	244			
**T**G	88	59	1.49	1.05-2.11	0.024
**TT**	6	1			
**rs6427202 (*F5*)**					
TT	99	103			
**C**T	191	165	1.32	1.04-1.66	0.021
**CC**	66	38			
**rs9332542 (*F5*)**					
**GG**	185	124			
A**G**	140	155	1.32	1.04-1.68	0.022
AA	32	28			
**rs6427199 (*F5*)**					
**GG**	146	97			
A**G**	156	161	1.27	1.02-1.59	0.036
AA	50	48			
**rs3093261 (*F10*)**					
CC	106	118			
**T**C	175	137	1.31	1.06-1.63	0.013
**TT**	77	51			
**rs2069948 (*EPCR*)**					
TT	103	118			
T**C**	173	125	1.25	1.01-1.54	0.037
**CC**	80	62			
