# Supplementary material for: Increased coagulation activity and genetic polymorphisms in the F5, F10 and EPCR genes are associated with breast cancer: a case-control study
Source: BMC Cancer. 2014 Nov 19;14:845. doi: 10.1186/1471-2407-14-845 (PMC4251949; doi:10.1186/1471-2407-14-845)
Supplement: Supplementary file 3 — Additional file 3: Table S2: The breast cancer associated SNPs stratified by hormone receptor status (ER and PR) and triple negative status (ER negative/PR negative/HER2 negative) (additive model in binary logistic regression). ORs determined with respect to the risk allele. Significant associations are shown in bold. (PDF 188 KB) [file 12885_2014_5043_MOESM3_ESM.pdf]

**Genotype distributions of the significant SNPs in TF pathway genes across ER, PR, HR, and triple negative status.**
ORs, 95% CI and *P*-values determined with respect to the risk allele (**bold**) using logistic regression (additive model). Alleles for the positive DNA strand (UCSC annotated) are shown.

| | Estrogen receptor (ER) status | | | | | | | | |
|---|---|---|---|---|---|---|---|---|---|
| **SNP** | **ER-negative cases (n)/ controls(n)** | **OR (95%CI)** | ***P*-value** | **ER-positive cases (n)/ controls (n)** | **OR (95%CI)** | ***P*-value** | **ER-negative cases(n)/ ER-positive cases(n)** | **OR (95%CI)** | ***P*-value** |
| **rs12120605 (*F5*)** | | | | | | | | | |
| GG | 40/244 | | | 202/244 | | | 40/202 | | |
| **T**G | 19/59 | **2.03** (1.14-3.61) | **0.016** | 64/59 | 1.4 (0.97-2.04) | 0.075 | 19/64 | 1.4 (0.81-2.42) | 0.228 |
| **TT** | 1/1 | | | 4/1 | | | 1/4 | | |
| **rs6427202 (*F5*)** | | | | | | | | | |
| TT | 12/103 | | | 81/103 | | | 12/81 | | |
| **C**T | 35/165 | **1.72** (1.12-2.64) | **0.014** | 140/165 | 1.24 (0.97-1.58) | 0.091 | 35/140 | 1.34 (0.89-2.03) | 0.164 |
| **CC** | 13/38 | | | 49/38 | | | 13/49 | | |
| **rs9332542 (*F5*)** | | | | | | | | | |
| **GG** | 31/124 | | | 139/124 | | | 31/139 | | |
| A**G** | 27/155 | **1.56** (1.01-2.39) | **0.044** | 105/155 | 1.23 (0.97-1.57) | 0.089 | 27/105 | 1.23 (0.81-1.86) | 0.327 |
| AA | 2/28 | | | 27/28 | | | 2/27 | | |
| **rs6427199 (*F5*)** | | | | | | | | | |
| **GG** | 29/97 | | | 108/97 | | | 29/108 | | |
| A**G** | 22/161 | 1.59 (0.99-2.54) | 0.054 | 121/161 | 1.27 (0.99-1.64) | 0.063 | 22/121 | 1.19 (0.76-1.85) | 0.449 |
| AA | 8/48 | | | 39/48 | | | 8/39 | | |
| **rs3093261 (*F10*)** | | | | | | | | | |
| CC | 10/118 | | | 88/118 | | | 10/88 | | |
| **T**C | 39/137 | **1.62** (1.09-2.40) | **0.018** | 123/137 | **1.26** (1.00-1.58) | **0.048** | 39/123 | 1.26 (0.85-1.86) | 0.254 |
| **TT** | 11/51 | | | 61/51 | | | 11/61 | | |
| **rs2069948 (*EPCR*)** | | | | | | | | | |
| TT | 19/118 | | | 78/118 | | | 19/78 | | |
| T**C** | 33/125 | 1.00 (0.68-1.46) | 0.998 | 129/125 | **1.27** (1.01-1.58) | **0.038** | 33/129 | 0.77 (0.87-1.93) | 0.208 |
| **CC** | 8/62 | | | 63/62 | | | 8/63 | | |

| | Progesterone receptor (PR) status | | | | | | | | |
|---|---|---|---|---|---|---|---|---|---|
| **SNP** | **PR-negative cases (n)/ controls(n)** | **OR (95%CI)** | ***P*-value** | **PR-positive cases (n)/ controls (n)** | **OR (95%CI)** | ***P*-value** | **PR-negative cases(n)/ PR-positive cases(n)** | **OR (95%CI)** | ***P*-value** |
| **rs12120605 (*F5*)** | | | | | | | | | |
| GG | 67/244 | | | 175/244 | | | 67/175 | | |
| **T**G | 30/59 | **1.96** (1.21-3.16) | **0.006** | 53/59 | 1.33 (0.90-1.97) | 0.150 | 30/53 | 1.44 (0.90-2.31) | 0.131 |
| **TT** | 2/1 | | | 3/1 | | | 2/3 | | |
| **rs6427202 (*F5*)** | | | | | | | | | |
| TT | 22/103 | | | 71/103 | | | 22/71 | | |
| **C**T | 57/165 | **1.57** (1.11-2.24) | **0.011** | 118/165 | 1.22 (0.94-1.58) | 0.135 | 57/118 | 1.26 (0.89-1.78) | 0.197 |
| **CC** | 20/38 | | | 42/38 | | | 20/42 | | |
| **rs9332542 (*F5*)** | | | | | | | | | |
| **GG** | 53/124 | | | 117/124 | | | 53/117 | | |
| A**G** | 39/155 | **1.73** (1.21-2.47) | **0.003** | 93/155 | 1.14 (0.89-1.47) | 0.296 | 39/93 | **1.45** (1.02-2.06) | **0.039** |
| AA | 6/28 | | | 23/28 | | | 6/23 | | |
| **rs6427199 (*F5*)** | | | | | | | | | |
| **GG** | 49/97 | | | 88/97 | | | 49/88 | | |
| A**G** | 39/161 | **1.55** (1.06-2.27) | **0.023** | 104/161 | 1.25 (0.95-1.63) | 0.107 | 39/104 | 1.2 (0.83-1.75) | 0.330 |
| AA | 10/48 | | | 37/48 | | | 10/37 | | |
| **rs3093261 (*F10*)** | | | | | | | | | |
| CC | 23/118 | | | 75/118 | | | 23/75 | | |
| **T**C | 57/137 | **1.44** (1.04-1.99) | **0.028** | 105/137 | **1.27** (1.00-1.61) | **0.048** | 57/105 | 1.11 (0.80-1.55) | 0.527 |
| **TT** | 19/51 | | | 53/51 | | | 19/53 | | |
| **rs2069948 (*EPCR*)** | | | | | | | | | |
| TT | 32/118 | | | 65/118 | | | 32/65 | | |
| T**C** | 52/125 | 1.02 (0.75-1.40) | 0.888 | 110/125 | **1.30** (1.03-1.65) | **0.025** | 52/110 | 0.77 (0.55-1.07) | 0.120 |
| **CC** | 15/62 | | | 56/62 | | | 15/56 | | |

| | Hormone receptor negative status (ER negative/PR negative) | | | | | |
|---|---|---|---|---|---|---|
| **SNP** | **ER/PR negative cases(n)/controls(n)** | **OR (95%CI)** | ***P*-value** | **ER/PR negative cases(n)/other cases(n)*** | **OR (95%CI)** | ***P*-value** |
| **rs12120605 (*F5*)** | | | | | | |
| GG | 39/244 | | | 39/203 | | |
| **T**G | 18/59 | **1.99** (1.11-3.56) | **0.021** | 18/65 | 1.36 (0.78-2.37) | 0.275 |
| **TT** | 1/1 | | | 1/4 | | |
| **rs6427202 (*F5*)** | | | | | | |
| TT | 11/103 | | | 11/82 | | |
| **C**T | 35/165 | **1.73** (1.12-2.67) | **0.014** | 35/140 | 1.34 (0.88-2.04) | 0.171 |
| **CC** | 12/38 | | | 12/50 | | |
| **rs9332542 (*F5*)** | | | | | | |
| **GG** | 30/124 | | | 30/140 | | |
| A**G** | 26/155 | 1.54 (0.99-2.37) | 0.053 | 26/106 | 1.18 (0.75-1.86) | 0.463 |
| AA | 2/28 | | | 2/27 | | |
| **rs6427199 (*F5*)** | | | | | | |
| **GG** | 28/97 | | | 28/109 | | |
| A**G** | 21/161 | 1.58 (0.98-2.55) | 0.058 | 21/122 | 1.21 (0.80-1.84) | 0.369 |
| AA | 8/48 | | | 8/39 | | |
| **rs3093261 (*F10*)** | | | | | | |
| CC | 10/118 | | | 10/88 | | |
| **T**C | 37/137 | **1.61** (1.08-2.41) | **0.020** | 37/125 | 1.26 (0.84-1.87) | 0.261 |
| **TT** | 11/51 | | | 11/61 | | |
| **rs2069948 (*EPCR*)** | | | | | | |
| TT | 18/118 | 1.02 (0.70-1.50) | 0.915 | 18/79 | 1.26 (0.84-1.88) | 0.269 |
| T**C** | 32/125 | | | 32/130 | | |
| **CC** | 8/62 | | | 8/63 | | |

*Cases with either ER negative/PR positive, ER positive/PR negative or ER positive/PR positive status

| | Triple negative status (ER negative/PR negative/HER2 negative) | | | | | |
|---|---|---|---|---|---|---|
| **SNP** | **ER/PR/HER2 negative cases(n)/ controls(n)** | **OR (95%CI)** | ***P*-value** | **ER/PR negative cases(n)/ other cases(n)*** | **OR (95%CI)** | ***P*-value** |
| **rs12120605 (*F5*)** | | | | | | |
| GG | 33/244 | | | 33/209 | | |
| **T**G | 16/59 | **2.11** (1.14-3.89) | **0.017** | 16/67 | 1.44 (0.81-2.58) | 0.216 |
| **TT** | 1/1 | | | 1/4 | | |
| **rs6427202 (*F5*)** | | | | | | |
| TT | 9/103 | | | 9/84 | | |
| **C**T | 29/165 | **1.90** (1.19-3.02) | **0.007** | 29/146 | 1.48 (0.95-2.32) | 0.084 |
| **CC** | 12/38 | | | 12/50 | | |
| **rs9332542 (*F5*)** | | | | | | |
| **GG** | 28/124 | | | 28/142 | | |
| A**G** | 21/155 | **1.68** (1.05-2.68) | **0.032** | 21/111 | 1.41 (0.86-2.31) | 0.179 |
| AA | 1/28 | | | 1/28 | | |
| **rs6427199 (*F5*)** | | | | | | |
| **GG** | 26/97 | | | 26/111 | | |
| A**G** | 16/161 | **1.88** (1.11-3.19) | **0.018** | 16/127 | 1.33 (0.84-2.09) | 0.223 |
| AA | 7/48 | | | 7/40 | | |
| **rs3093261 (*F10*)** | | | | | | |
| CC | 10/118 | | | 10/88 | | |
| **T**C | 32/137 | 1.44 (0.94-2.19) | 0.095 | 32/130 | 1.09 (0.72-1.67) | 0.680 |
| **TT** | 8/51 | | | 8/64 | | |
| **rs2069948 (*EPCR*)** | | | | | | |
| TT | 16/118 | | | 16/81 | | |
| T**C** | 28/125 | 1.03 (0.68-1.55) | 0.883 | 28/134 | 1.33 (0.87-2.06) | 0.191 |
| **CC** | 6/62 | | | 6/65 | | |

*Cases with either ER negative/PR positive/HER2 positive, ER negative/PR positive/HER2 negative, ER positive/PR negative/HER2 positive, ER positive/PR negative/HER2 negative, ER positive/PR positive/Her2 positive, ER positive/PR positive/Her2 negative, ER negative/PR negative/HER2 positive status.
