# Supplementary material for: Increased coagulation activity and genetic polymorphisms in the F5, F10 and EPCR genes are associated with breast cancer: a case-control study
Source: BMC Cancer. 2014 Nov 19;14:845. doi: 10.1186/1471-2407-14-845 (PMC4251949; doi:10.1186/1471-2407-14-845)
Supplement: Supplementary file 4 — Additional file 4: Table S3: Plasma levels of hemostatic parameters in cases and controls. Median values with IQR shown in brackets. (PDF 90 KB) [file 12885_2014_5043_MOESM4_ESM.pdf]

Plasma levels of hemostatic parameters in cases and controls.
Median values with IQR shown in brackets.

	**Cases** (n=366)	**Controls** (n=273)	
	**Median** (IQR)	**Median** (IQR)	***P*-value**
**Coagulation activity:**			
CAT-assay variables:			
ETP (%)	94.9 (19.8)	94.1 (19.1)	n.s
Lag time (%)	98.6 (21.4)	100.0 (16.5)	$2.9*10^{-17}$
Peak (%)	98.4 (17.9)	86.7 (15.4)	$3.3*10^{-26}$
ttPeak (%)	93.8 (12.8)	107.9 (15.5)	$7.1*10^{-36}$
APC resistance (nAPC-sr)			
FV Leiden non-carriers	0.75 (0.81)	0.39 (0.35)	$2.2*10^{-19}$
FV Leiden carriers	3.73 (1.02)	2.20 (1.12)	$6.6*10^{-5}$
D-dimer (ng/mL)	300.0 (189.3)	275.5 (146.9)	0.015
**Coagulation inhibitors:**			
AT (%)	101.5 (16.9)	107.7 (11.7)	$3.3*10^{-11}$
Protein C (%)	109.4 (31.1)	101.9 (23.5)	0.002
Protein S (%)	93.9 (20.8)	87.6 (21.2)	$3.6*10^{-6}$
Free TFPI (ng/mL)	10.1 (4.8)	10.6 (4.1)	n.s

Peak= peak thrombin
ttPeak= time to peak thrombin
nAPC-sr= normalised APC sensitivity ratio
AT= antithrombin
(%) describes activity as compared to PNP
IQR; interquartile range (75th percentile-25th percentile)
