# Supplementary material for: Increased coagulation activity and genetic polymorphisms in the F5, F10 and EPCR genes are associated with breast cancer: a case-control study
Source: BMC Cancer. 2014 Nov 19;14:845. doi: 10.1186/1471-2407-14-845 (PMC4251949; doi:10.1186/1471-2407-14-845)
Supplement: Supplementary file 5 — Additional file 5: Table S4: ORs and P-values for hemostatic parameters stratified by hormone receptor status (ER and PR) and triple negative status (ER negative/PR negative/HER2 negative). (PDF 99 KB) [file 12885_2014_5043_MOESM5_ESM.pdf]

ORs and *P*-values for hemostatic hemostatic parameters stratified by hormone receptor status (ER and PR) and triple negative status (ER negative/PR negative/HER2 negative)

	**Estrogen receptor (ER) status**								
**Parameter**	**ER-negative cases (n)/ controls (n)**	**OR (95% CI)**	***P* (unadj.)**	**ER-positive cases (n)/ controls (n)**	**OR (95% CI)**	***P* (unadj.)**	**ER-negative cases(n)/ ER-positive cases(n)**	**OR (95% CI)**	***P* (unadj.)**
**Coagulation activity*:***									
CAT-assay parameters:									
Lagtime									
>10th percentile	31/246	Ref.	Ref.	165/246	Ref.	Ref.	31/165	Ref.	Ref.
<10th percentile	24/27	7.05 (3.63-13.71)	<0.001	96/27	5.30 (3.31-8.48)	<0.001	24/96	1.33 (0.74-2.40)	0.342
Peak									
<90th percentile	32/246	Ref.	Ref.	156/246	Ref.	Ref.	32/156	Ref.	Ref.
>90thpercentile	23/27	6.55 (3.36-12.76)	<0.001	105/27	6.13 (3.84-9.79)	<0.001	23/105	1.07 (0.59-1.93)	0.827
ttPeak									
>10th percentile	29/249	Ref.	Ref.	159/249	Ref.	Ref.	29/159	Ref.	Ref.
<10th percentile	26/24	9.30 (4.74-18.27)	<0.001	102/24	6.66 (4.09-10.83)	<0.001	26/102	1.40 (0.78-2.51)	0.262
APC resistance									
FVL non-carriers									
<90th percentile	29/228	Ref.	Ref.	145/228	Ref.	Ref.	29/145	Ref.	Ref.
>90thpercentile	24/25	7.55 (3.82-14.90)	<0.001	98/25	6.16 (3.79-10.02)	<0.001	24/98	1.22 (0.67-2.23)	0.507
FVL carriers									
<90th percentile	0/18	Ref.	Ref.	3/18	Ref.	Ref.	0/3	Ref.	Ref.
>90thpercentile	2/2	NA	NA	15/2	45.00 (6.62-305.69)	<0.001	2/15	NA	NA
D-dimer									
<90th percentile	48/246	Ref.	Ref.	217/246	Ref.	Ref.	48/217	Ref.	Ref.
>90thpercentile	10/27	1.90 (0.86-4.18)	0.111	49/27	2.06 (1.24-3.41)	0.005	10/49	0.92 (0.44-1.95)	0.833
**Coagulation inhibitor:**									
AT									
>10th percentile	38/246	Ref.	Ref.	155/246	Ref.	Ref.	38/155	Ref.	Ref.
<10th percentile	19/27	4.56 (2.31-8.98)	<0.001	107/27	6.29 (3.94-10.04)	<0.001	19/107	0.72 (0.40-1.32)	0.295

Parameter	Progesterone receptor (PR) status								
	**PR-negative cases(n)/ controls (n)**	**OR (95% CI)**	***P* (unadj.)**	**PR-positive cases (n)/ controls (n)**	**OR (95% CI)**	***P* (unadj.)**	**PR-negative cases (n)/ PR-positive cases (n)**	**OR (95% CI)**	***P* (unadj.)**
**Coagulation activity*:***									
CAT-assay parameters:									
Lagtime									
>10th percentile	56/246	Ref.	Ref.	140/246	Ref.	Ref.	56/140	Ref.	Ref.
<10th percentile	37/27	6.02 (3.39-10.70)	<0.001	83/27	5.40 (3.34-8.74)	<0.001	37/83	1.11 (0.68-1.83)	0.669
Peak									
<90th percentile	60/246	Ref.	Ref.	128//246	Ref.	Ref.	60/128	Ref.	Ref.
>90thpercentile	33/27	5.01 (2.80-8.96)	<0.001	95/27	6.76 (4.19-10.90)	<0.001	33/95	0.74 (0.45-1.22)	0.241
ttPeak									
>10th percentile	54/249	Ref.	Ref.	134/249	Ref.	Ref.	54/134	Ref.	Ref.
<10th percentile	39/24	7.49 (4.16-13.48)	<0.001	89/24	6.89 (4.19-11.33)	<0.001	39/89	1.09 (0.67-1.78)	0.738
APC resistance									
FVL non-carriers									
<90th percentile	65/228	Ref.	Ref.	164/228	Ref.	Ref.	65/164	Ref.	Ref.
>90thpercentile	23/25	3.23 (1.72-6.06)	<0.001	44/25	2.45 (1.44-4.16)	0.001	23/44	1.32 (0.74-2.36)	0.350
FVL carriers									
<90th percentile	0/18	Ref.	Ref.	3/18	Ref.	Ref.	0/3	Ref.	Ref.
>90thpercentile	5/2	NA	NA	12/2	36.00 (5.21-248.66)	<0.001	5/12	NA	NA
D-dimer									
<90th percentile	79/246	Ref.	Ref.	186/246	Ref.	Ref.	79/186	Ref.	Ref.
>90thpercentile	16/27	1.85 (0.95-3.60)	0.072	43/27	2.11 (1.26-3.53)	0.004	16/43	0.88 (0.47-1.65)	0.681
**Coagulation inhibitor:**									
AT									
>10th percentile	61/246	Ref.	Ref.	132/246	Ref.	Ref.	61/132	Ref.	Ref.
<10th percentile	32/27	4.78 (2.67-8.57)	<0.001	94/27	6.49 (4.03-10.46)	<0.001	32/94	0.74 (0.45-1.22)	0.234

Parameter	Hormone receptor negative status (ER negative/PR negative)		
	**ER/PR negative cases (n)/ other cases* (n)**	**OR (95% CI)**	***P* (unadj.)**
**Coagulation activity*:***			
CAT-assay parameters:			
Lagtime			
>10th percentile	31/165	Ref.	Ref.
<10th percentile	22/98	1.19 (0.66-2.18)	0.561
Peak			
<90th percentile	31/157	Ref.	Ref.
>90thpercentile	22/106	1.05 (0.58-1.91)	0.870
ttPeak			
>10th percentile	29/159	Ref.	Ref.
<10th percentile	24/104	1.27 (0.70-2.29)	0.438
APC resistance			
FVL non-carriers			
<90th percentile	29/145	Ref.	Ref.
>90thpercentile	22/100	1.10 (0.60-2.02)	0.759
FVL carriers			
<90th percentile	0/3	Ref.	Ref.
>90thpercentile	2/15	NA	NA
D-dimer			
<90th percentile	46/219	Ref.	Ref.
>90thpercentile	10/49	0.97 (0.46-2.06)	0.940
**Coagulation inhibitor:**			
AT			
>10th percentile	36/157	Ref.	Ref.
<10th percentile	19/107	0.77 (0.42-1.42)	0.410

*****Cases with either ER negative/PR positive, ER positive/PR negative or ER positive/PR positive status

Parameter	Triple negative status (ER/PR/HER2 negative)		
	**Triple negative cases (n)/ other cases* (n)**	**OR (95% CI)**	***P* (unadj.)**
**Coagulation activity*:***			
CAT-assay parameters:			
Lagtime			
>10th percentile	25/171	Ref.	Ref.
<10th percentile	20/100	1.37 (0.72-2.59)	0.335
Peak			
<90th percentile	26/162	Ref.	Ref.
>90thpercentile	19/109	1.09 (0.57-2.06)	0.800
ttPeak			
>10th percentile	24/164	Ref.	Ref.
<10th percentile	21/107	1.34(0.71-2.53)	0.364
APC resistance			
FVL non-carriers			
<90th percentile	23/151	Ref.	Ref.
>90thpercentile	20/102	1.29 (0.67-2.47)	0.446
FVL carriers			
<90th percentile	0/3	Ref.	Ref.
>90thpercentile	2/15	NA	NA
D-dimer			
<90th percentile	40/225	Ref.	Ref.
>90thpercentile	8/51	0.88 (0.39-2.00)	0.764
**Coagulation inhibitor:**			
AT			
>10th percentile	32/161	Ref.	Ref.
<10th percentile	15/111	0.68 (0.35-1.31)	0.251

*Cases with either ER negative/PR positive/HER2 positive, ER negative/PR positive/HER2 negative, ER positive/PR negative/HER2 positive, ER positive/PR negative/HER2 negative, ER positive/PR positive/Her2 positive, ER positive/PR positive/Her2 negative, ER negative/PR negative/HER2 positive status.
